# Metabolomic phenotyping of a cloned pig model

**DOI:** 10.1186/1472-6793-11-14

**Published:** 2011-08-22

**Authors:** Morten R Clausen, Kirstine L Christensen, Mette S Hedemann, Ying Liu, Stig Purup, Mette Schmidt, Henrik Callesen, Jan Stagsted, Hanne C Bertram

**Affiliations:** 1 Department of Food Science, Science and Technology, Aarhus University, Aarslev, Denmark; 2 Department of Animal Health and Bioscience, Science and Technology, Aarhus University, Tjele, Denmark; 3 Department of Genetics and Biotechnology, Science and Technology, Aarhus University, Tjele, Denmark; 4 Veterinary Reproduction and Obstetrics, Faculty of Life Sciences, University of Copenhagen, Frederiksberg C, Denmark; 5 Department of Food Science, Science and Technology, Aarhus University, Tjele, Denmark

## Abstract

**Background:**

Pigs are widely used as models for human physiological changes in intervention studies, because of the close resemblance between human and porcine physiology and the high degree of experimental control when using an animal model. Cloned animals have, in principle, identical genotypes and possibly also phenotypes and this offer an extra level of experimental control which could possibly make them a desirable tool for intervention studies. Therefore, in the present study, we address how phenotype and phenotypic variation is affected by cloning, through comparison of cloned pigs and normal outbred pigs.

**Results:**

The metabolic phenotype of cloned pigs (n = 5) was for the first time elucidated by nuclear magnetic resonance (NMR)-based metabolomic analysis of multiple bio-fluids including plasma, bile and urine. The metabolic phenotype of the cloned pigs was compared with normal outbred pigs (n = 6) by multivariate data analysis, which revealed differences in the metabolic phenotypes. Plasma lactate was higher for cloned vs control pigs, while multiple metabolites were altered in the bile. However a lower inter-individual variability for cloned pigs compared with control pigs could not be established.

**Conclusions:**

From the present study we conclude that cloned and normal outbred pigs are phenotypically different. However, it cannot be concluded that the use of cloned animals will reduce the inter-individual variation in intervention studies, though this is based on a limited number of animals.

## Background

Use of animal models in research related to human health and nutrition is common practice for example in dietary intervention studies. There are several reasons for using animal models. Firstly, the access to several bio-fluids and organs is possible. Secondly, it is easier to control animals than humans and to secure compliance to the experimental diet. This should lead to smaller inter-individual differences which are necessary for showing effects of the compound/diet under investigation. For this reason we hypothesize that a cloned animal model would be beneficial for intervention studies, as they are expected to provide a more controlled and repeatable experimental system that requires fewer animals compared with outbred lines.

In particular, the pig has become a widely used model, since pigs from a nutritional aspect are comparable to humans [1,2], and their lipoprotein profile and metabolism are similar to that of humans [3-6]. Recently, a cloned pig model was used as a model for studying atherosclerosis [7]. However, the use of cloned animals in nutrition studies is still in its opening stage, and a more comprehensive elucidation of the usefulness of a cloned pig model in these types of studies is needed. Cloned pigs will have identical DNA sequences and in principle identical phenotypes. However, in the cloning process, single somatic cell nuclei are introduced into each their enucleated oocyte containing mitochondrial DNA, so a small subset of mitochondrial proteins will be of maternal origin [8]. Additionally, the somatic cell nuclei may have different epigenetic constitution, i.e. although the DNA sequences are identical, the methylation degree may vary between nuclei which could lead to differences in expression of certain genes and therefore to variable phenotypes. Consequently, the phenotypic variation of cloned pigs is so far unknown, and there is only limited data available in the literature [9,10].

The phenotype, i.e. an individual's observable traits, is expressed in the metabolome. Proton nuclear magnetic resonance (^1^H NMR) spectroscopy is probably the most widely applied technique for studying the metabolome based on bio-fluids, and has been used for metabolic phenotyping of humans [11]. The porcine metabolome has also been subject to investigations, and the potential of NMR-based metabolomics for elucidating the biochemical effects of dietary components such as rye versus wheat fibers [12,13], and arginine supplementation [14] as well as for studying the impact of birth weight on the plasma metabolome has been established [15]. However, no metabolomic investigations have so far been reported on cloned pigs. The importance of such a characterization is further underlined by the widespread use of pigs as a model in studies of cardiovascular disease, diabetes, and the metabolic syndrome [2], since the usefulness of such a model must rely on similarities in phenotype and in response to experimental treatments.

Therefore the aim of the present study was to elucidate the phenotype of a cloned pig model by characterization of multiple bio-fluids (urine, plasma and bile) using NMR-based metabolomics by comparison with outbred control pigs.

## Results

### Multivariate data analysis of bio-fluids

**Figure 1:**
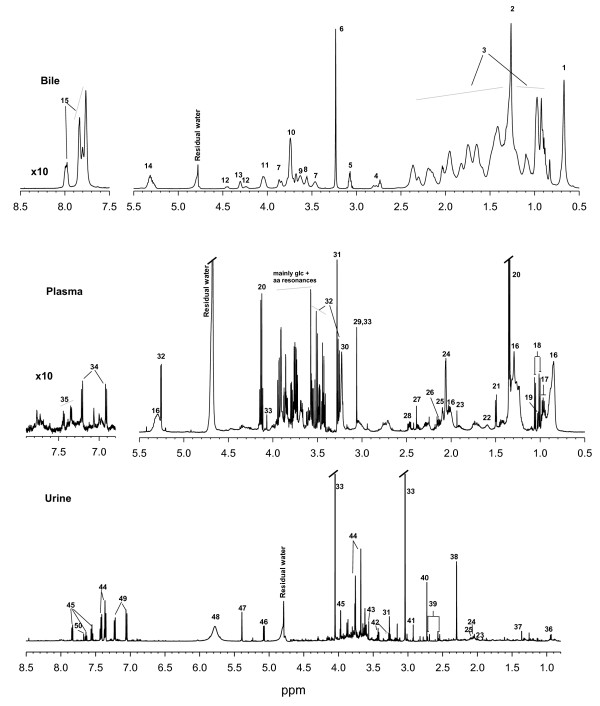
**NMR spectra and assignments**. Representative ^1^H NMR spectra of bile, plasma and urine obtained from a cloned pig. All spectra were acquired on a 600 MHz spectrometer. Less abundant amino acids (Glu, Gln, Lys, and Arg) with complex spectra could not be unambiguously assigned in the spectral region 1.6 - 2.6 ppm in urine. Assignments: **1**, bile acids, cholesterol; **2**, lipids (-CH_2_-); **3**, bile acids cholesterol, lipids; **4**, bisallyllic protons; **5**, conjugated taurine; **6**, choline or phosphatidylcholine (PC) (-N-(CH_3_)_3_); **7**, glycine/taurine conjugated cholate (CA), glycine/taurine conjugated chenodeoxy cholate (CDCA); **8**, Conjugated deoxycholate (H-3β), conjugated taurine (H-25); **9**, PC (CH_2_-N); **10**, Conjugated glycine (H-25); **11**, conjugated cholate and deoxycholate, PC-glycerol (3-CH_2_); **12**, PC-glycerol (1-CH); **13**, PC (-O-CH2-); **14**, CH = CH, cholesterol (6-CH), PC-glycerol (2-CH); **15**; Conj bile acids (-NH-); **16**, LDL/VLDL; **17**, leucine; **18**, valine; **19**, isoleucine; **20**, lactate; **21**, alanine; **22**, adipate; **23**, acetate; **24**, N-acetyl glycoproteins; **25**, O-acetyl glycoproteins; **26**, glutamine/glutamate; **27**, pyruvate; **28**, glutamate; **29**, creatine; **30**, choline; **31**, trimethylamine-N-oxide (TMAO); **32**, glucose; **33**, creatinine; **34**, tyrosine; **35**, phenylalanine; **36**, isovaleraldehyde; **37**, α-hydroxyisobutyrate; **38**, unknown; **39**, citrate; **40**, dimethylamine; **41**, trimethyl amine; **42**, taurine; **43**, glycine; **44**, phenylacetylglycine; **45**, hippurate; **46**, unknown; **47**, alantoin; **48**, urea; **49**, unknown; **50**, guanine. For further information about identified metabolites, please refer to Additional file 1

**Figure 2:**
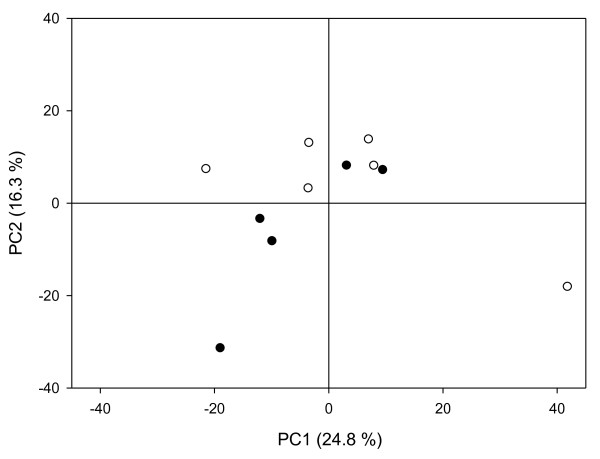
**Urine PCA**. PCA score plots of urine samples from cloned pigs (closed symbols) and control pigs (open symbols). n = 11. Cumulated explained variance, *R^2^X *= 0.57, Cumulated cross validated explained variance, *Q^2^*(cum) = -0.17 for the three component model made from normalized data.

**Figure 3:**
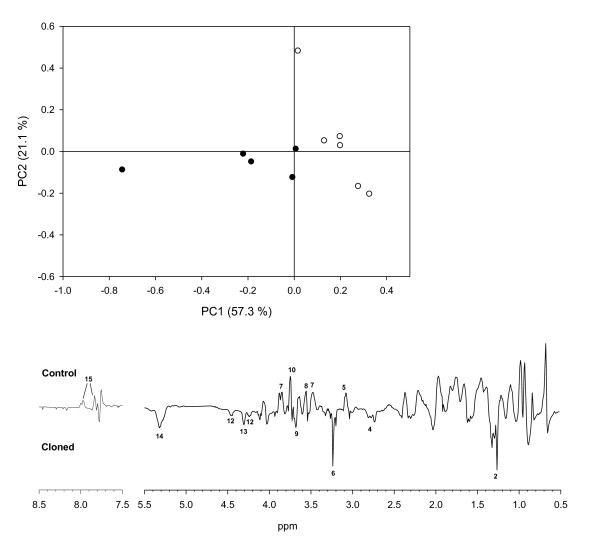
**Bile PCA**. PCA score plot of bile samples from cloned pigs (closed symbols) and control pigs (open symbols) (a) and PC1 loading plot (b). n = 11. *R^2^X *= 0.57, *Q^2^*(cum) = 0.36 for the one component model. For visualization principal component 2 is shown in the score plot. Major discriminatory resonances are marked with numbers that correspond to the identifications in figure 1.

**Figure 4:**
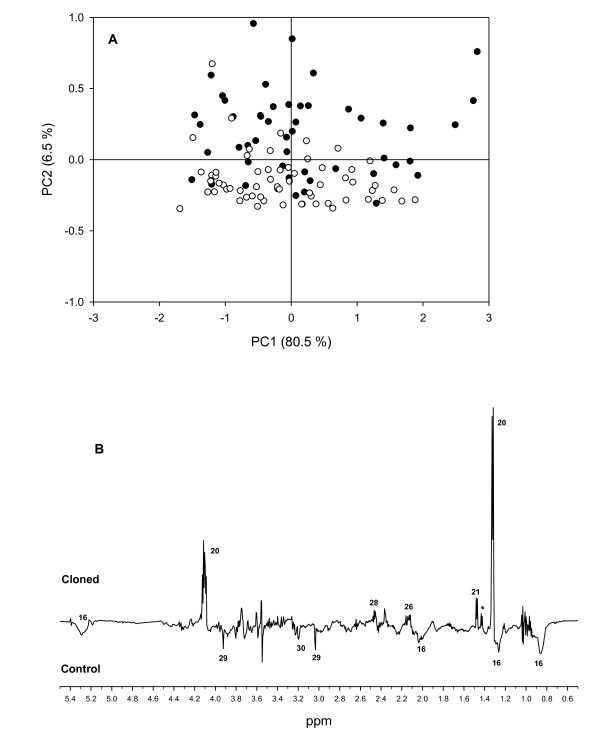
**Plasma PCA**. PCA score plot of plasma samples from cloned pigs (closed symbols) and control pigs (open symbols) (a) and PC2 loading plot (b). n = 112. *R^2^X *= 0.97, *Q^2^*(cum) = 0.74 for the 10 component model. Major discriminatory resonances are marked with numbers that correspond to the identifications in figure 1. Asterisk denotes presumed threonine, which could not be resolved in the 1-D NMR spectrum.

Representative ^1^H NMR spectra obtained for plasma, urine and bile are shown in Figure 1 (also see Additional File 1 and Additional file 2 for all data files). The NMR spectra were assigned by comparison with established libraries reported in the literature [16], the Human Metabolome Data Base (HMDB) [17], by comparison with previous studies [18,19], and with pure standards. Potential differences in the NMR metabolite profiles between cloned pigs and control pigs were investigated using PCA. For plasma and bile a tendency for grouping of cloned pigs and control pigs was observed, whereas for urine no grouping was observed (Figures 2, 3 and 4). Furthermore, while the cross-validated predictive ability of the PCA models was good for plasma and bile, the cross-validated predictive ability was poor for the PCA model obtained on urine samples (Table 1 and Figure 2), and interpretation of these data would probably require a larger set of samples.

**Table 1:** PCA models for NMR spectral data

Bio-fluid	Normalization	n	Number of components	R^2^X	Q^2 ^(cum)
Bile	+	11	1	0.57	0.36
Bile	-^a^	11	2	0.40	0.37

Urine	+	11	3	0.57	-0.17
Urine	-	11	2	0.39	0.12

Plasma	+	110	10	0.97	0.74
Plasma	-	110	10	0.95	0.91

Summary of PCA models of all biofluids, with and without normalization

^a^First component was used to describe the variation induced by one outlier. This outlier was included in the analysis, since the second component, which described the difference between groups, was not affected by it.

For bile and plasma, PCA resulted in differentiation between cloned and control pigs irrespective of normalization. Classification of plasma, however, was due to a global difference in metabolite concentrations covering all resonances. No explanation could be elucidated for this difference, and thus, this model did not reveal any information on metabolic differences between the two groups. For bile, performances of the normalized and non-normalized models were similar (Table 1), and inspection of loadings showed that they were super-imposable, thus leading to the same conclusions (not shown). Accordingly, for data in the present study normalization had no effect on bile samples, whereas normalization of plasma data had a prominent effect. All multivariate data analysis is therefore based on normalized data.

### Bile

Loadings from PCA revealed that for bile, multiple signals in the NMR spectra contributed to the differentiation of cloned pigs and control pigs (Figure 3). Bile from cloned pigs was characterised by higher intensities of signals assigned to choline (-N-(CH_3_)_3_, 3.22 ppm), phosphatidyl choline (PC) glycerol moiety (4.2 - 4.5 ppm), and PC (-CH_2_-N 3.68 ppm)). Unsaturated lipids, probably also related to phospholipids, and total lipids, were also more abundant in bile from cloned pigs. Signals from conjugated cholate and conjugated chenodeoxy cholate (7.72 - 8.05 ppm, 3.72 ppm; 3.56 ppm; 3.07 ppm) as well as their un-conjugated forms (3.46 ppm; 3.86 ppm) were consistently more abundant in bile from control pigs compared with cloned pigs.

### Plasma

**Figure 5:**
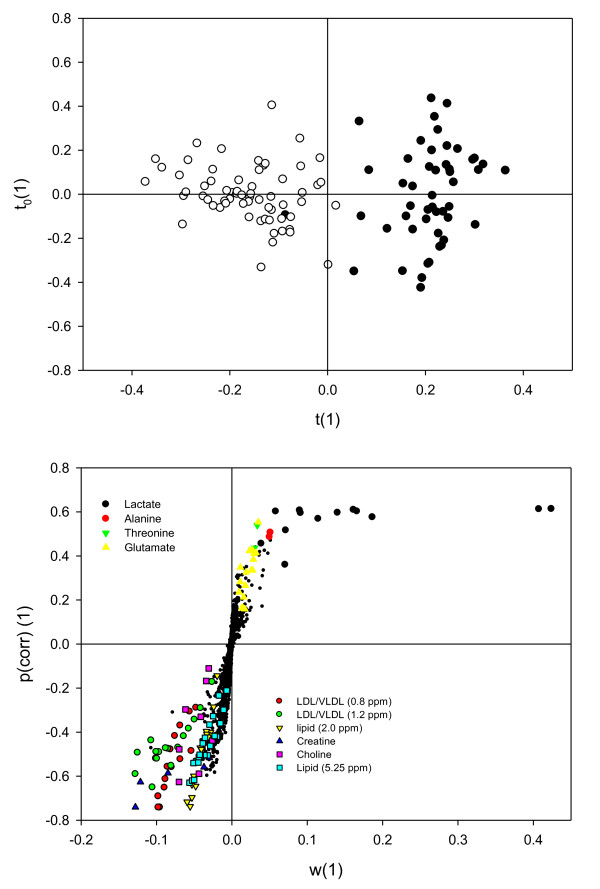
**Plasma O-PLS**. O-PLS score plot of all plasma samples from cloned pigs (closed symbols) and control pigs (open symbols) (a) and S-plot for plasma metabolites (b). R^2^X = 0.72, R^2^Y = 0.83, Q^2^(cum) = 0.65. Variables with high discriminatory ability are identified in the S-plot and metabolites related to these variables are enlarged and coded with colours according to the legend. For clarity other variables are shown as dots.

The PCA model of the NMR spectra of plasma samples included samples from all sampling days, thus including 9 - 11 samples of plasma from each individual pig. Manual inspection of PC1 to PC4 of the obtained model did not reveal any clustering according to sampling date, and this aspect was not investigated further. As shown in Figure 4, a clustering between cloned pigs and control pigs along PC2 could be observed and the loadings for this principal component indicates that this is mainly due to differences in lactate concentrations (1.33 ppm and 4.11 ppm) and to a minor extent signals from lipids, and a number of amino acids. Since multiple sampling was carried out an O-PLS-DA model could be constructed and cross-validated without risk of over-fitting data. This analysis confirmed that the difference between cloned pigs and control pigs should be ascribed to a higher concentration of lactate in plasma from cloned pigs compared with control pigs (Figure 5a and 5b). The constructed S-plot (Figure 5b) revealed that also alanine (1.48 ppm), threonine (1.42 ppm), and glutamate (2.45 ppm) were more abundant in plasma from cloned pigs, whereas in plasma from control pigs signals from lipoproteins (0.80 ppm; 1.20 ppm; 2.00 ppm; 5.25 ppm), creatine (3.03 ppm; 3.92 ppm), and choline (3.19 ppm) were most abundant. Relative integrals of these metabolites are presented in Table 2 and confirm these conclusions, and indicate that lactate and creatine are the most important discriminatory compounds.

**Table 2:** Relative areas of selected metabolites in plasma determined by NMR integrals

Compound	Observed δ, ppm	Clone			Control		
Alanine	1.48	7.7	±	0.5	6.8	±	0.2
Choline	3.21	16	±	1.2	19	±	2.0
Creatine	3.92	3.1	±	0.4	5.2	±	0.7
Glutamate	2.45	8.2	±	0.2	7.5	±	0.1
Lactate	4.11	26	±	4.7	15	±	2.0
Lipid	5.30	18.5	±	0.9	22	±	1.2
VLDL/LDL	0.85	64	±	4.6	77	±	3.5
VLDL/LDL	1.25	89	±	5.2	109	±	3.4

Results are reported as mean ± standard deviation. Cloned pigs (n = 5) and control pigs (n = 6). All metabolites were significantly different between the two groups with p < 0.001.

### Effect of cloning on inter-individual variation

The most intense signals were integrated, and the variances were calculated for each integral, in order to elucidate the inter-individual variation for the cloned and control pigs. The ratios between the variances for cloned and for control pigs were then determined. These ratios provide a measure that can easily establish which group has the largest variation, as a value larger than one reflects a *higher *variation for cloned pigs than for control pigs, while a value smaller than one reflects a *lower *variation for cloned pigs than for control pigs. Only well-resolved peaks were used for this analysis, and consequently 10, 7, and 15 signals were integrated in bile, plasma and urine respectively (Table 3 and Additional file 3). Only three significant differences in variance between cloned and control pigs were observed (bile conjugated cholate/chenodeoxy cholate, plasma valine, and urine hippurate), and could thus not support the hypothesis that cloned pigs are less variable than control pigs. The numbers of ratios above and below one were 10 and 14 respectively, after removing values that originated from the same compound (Table 3). Under the hypothesis that the variance in cloned and control animals is similar (p = 0.5) this distribution of ratios was not found to be significantly different from 50:50 and indicated a random distribution of variances.

**Table 3:** Variances calculated for integrals from bile, plasma, and urine

				Variance		
					
Peak number	Biofluid	Observed δ, ppm	Signal range	Clone	Control	v clone/v control
**1**	Bile	0.62	87 - 102	5, 98	39.1	0.15
**4**	Bile	2.75	2.9 - 14.7	1.60	0.89	1.80
**7**	Bile	3.45	8.5 - 13.5	0.60	5.82	0.048a
**15**	Bile	7.80	13.8 - 18.0	0.19	1.26	0.15
	Bile	7.98	1.8 - 4.9	0.57	2.15	0.26
**5**	Bile	3.07	5.9 - 16.8	7.03	12.5	0.56
**6, 12**	Bile	4.30	3.7 - 8.5	0.94	0.74	1.27
		4.42	0.5 - 3.8	0.049	0.036	1.38
		3.25	12 - 53	15.2	7.05	2.16
**23**	Plasma	1.91 (s)	0.005 - 0.05	5.40E-5	4.47E-5	1.21
**21**	Plasma	1.47 (d)	0.006 - 0.1	0.00038	0.00037	1.02
**33**	Plasma	4.04 (s)	0 - 0.03	2.77E-5	4.38E-5	0.63
**20**	Plasma	4.10 (q)	0.03 - 0.7	0.013	0.017	0.78
**27**	Plasma	2.36 (s)	0.003 - 0.04	4.54E-5	3.37E-5	1.35
**34**	Plasma	7.18 (d)	0.003 - 0.03	3.12E-5	3.32E-5	0.94
**18**	Plasma	1.03 (d)	0.004 - 0.3	0.0018	0.00038	4.58a
**21**	Urine	1.48 (d)	0.82 - 7.44	2.2	2.5	0.87
**47**	Urine	5.39 (s)	1.89 - 31.4	112	53	2.11
**33**	Urine	3.04 (s)	44 - 497	2.4E4	6.5E3	3.75
	Urine	4.05 (s)	31 - 349	1.2E4	3.2E3	3.69
**45**	Urine	7.84 (d)	3.6 - 40	138	61	2.26a
		7.55 (t)	3.7 - 47	185	67	2.85a
		3.97 (m)	8.0 - 87	524	178	2.94a
		7.64 (t)	8.1 - 28	64	19	3.44a
**36**	Urine	0.94 (d)	2.8 - 18	23	14	1.61
**37**	Urine	1.36 (s)	2.9 - 11	7.2	4.0	1.80
**44**	Urine	7.37 (m)	1.8 - 17	5.7	30.1	0.19
**46**	Urine	7.23 (d)	2.2 - 68	547	181	3.02
	Urine	7.06 (d)	0.7 - 60	514	146	3.52
**49**	Urine	5.08 (d)	0.0 - 22.1	79	15	5.20
**48**	Urine	5.78	78 - 643	2.0E4	1.2E4	1.62

Variances based on normalized urine spectra and raw bile and plasma spectra. For each metabolite the ratios between cloned pig variances and control pig variances are shown. Peak numbers refer to the legend in Figure 1

a, significant values.

## Discussion

Cloned animals are expected to be more homogenous than outbred lines and could therefore represent a good model for research purposes where a small inter-individual variation is desired. However, only sparse data are available about the phenotypic variation of cloned pigs [9,10]. The present study for the first time reports a metabolomic phenotyping of cloned pigs. Using NMR-based metabolomics we have shown that the metabolite profile of plasma and bile, but not urine, differed for cloned pigs and normal outbred pigs. In fact, the bile and plasma concentrations of multiple metabolites differed suggesting that the cloned pigs had an altered metabolic phenotype compared with the control outbred pigs.

For plasma samples it was identified that lactate levels were higher in cloned pigs than in normal outbred pigs. Alanine and glutamate, which are both linked to the citrate cycle, were also found in higher concentrations in cloned pigs. The reason for an increased plasma lactate remains unknown. In a study on heifers, cloned animals were found to have higher oxidative metabolism than control animals as assessed by isocitrate dehydrogenase, cytochrome-c oxidase and beta-hydroxyacyl-CoA dehydrogenase activities in muscle biopsies [20]. However, the increased plasma lactate levels observed in this study indicate higher anaerobic metabolism. Consequently, the increased lactate levels in the cloned pigs are probably a consequence of another mechanism. Oocytes and embryos are inevitably exposed to oxidative stress generated by reactive oxygen species during in vitro culture [21], and this might have impact on stress sensitivity post-natally in the cloned pig. The higher plasma lactate levels might therefore reflect higher stress sensitivity in cloned pigs compared with normal pigs due to the in vitro cloning procedure.

Plasma lipid and lipoprotein signals were higher in the control group as compared to the cloned group, which may be related to differences in the regulation of lipoprotein circulation. Likewise creatine, which is involved in cellular energy production, was elevated in control pigs. Thus, several metabolites indicate that the response of cloned pigs and control pigs to a dietary intervention is not the same, and this could affect the applicability in relation to human nutrition.

Furthermore, differences in bile composition, which is also under genetic control, could affect lipid metabolism [22]. The relative content of bile acids and phospholipids affects the micelle surface and core composition in the small intestine, and thus probably has an impact on the absorption of dietary lipids [23]. Increasing amounts of phospholipids have also been shown to reduce the critical micelle concentration of bile salts, thus affecting the number of micelles [24,25]. In the present study high amounts of bile acids in the bile were associated with an increased content of lipids in plasma, though a direct causal connection could not be established. Studies with rodents have shown that an increase in plasma bile acids induced by diet, reduce liver VLDL secretion and prevent elevated serum triacylglycerol concentration [26]. These findings seem opposite to our data and further work is needed to elucidate the physiological role of production and circulation of bile acids, which also appears to be important for weight regulation [27].

In order to elucidate the potential of a cloned pig model, it is also important to consider the inter-individual variation. Consequently, for all bio-fluids the variance of individual metabolites was calculated and compared with the variance for control outbred pigs. This analysis did not reveal a significant difference in variation between the two groups. Consequently, based on the present study it cannot be concluded that the inter-individual variation in the metabolic phenotype is smaller for cloned pigs compared with normal outbred pigs. While a standardized genotype does not seem to affect the inter-individual variation, the gut-microbiota is known to affect host phenotype [19]. In the present study no systematic effect of gut-microbiota was expected since all animals received the same diet, and no difference in the contents of metabolites originating from microbial fermentation (e.g. acetic acid, butyric acid) could be demonstrated in the present study. Fluctuations in the host metabolome could arise, however, as a result of the complex interactions between nutrition, immune function, and gut-microbiota, and therefore the gut-microbiomes of cloned and normal pigs are currently being analyzed and will be the subject of additional papers. Therefore, when a low inter-individual variation between subjects is required, a standardization of the gut-microbiota might be more important than standardization of the genotype [28]. However, the present study only included a limited number of animals, and further studies with larger numbers of subjects are needed to substantiate these results. In addition the metabolites included in the analysis were chosen among the most intense resonances, which may introduce a bias. Less abundant metabolites, which are not easily detected by NMR-based metabolomics, are currently being analyzed by LC-MS-based metabolomics and will be the subject of a subsequent paper

## Conclusions

In conclusion, despite the limited number of animals, the present metabolomic study on multiple bio-fluids clearly indicated alterations in the metabolic phenotype of cloned pigs compared with control pigs, and this should be taken into consideration when cloned animals are used as model animals. In addition, the present NMR-based analyses of plasma, bile and urine could not demonstrate a smaller inter-individual variation in cloned pigs compared with control pigs.

## Methods

### Animals and sampling

All experimental procedures involving animals were approved by the Danish Animal Experimental Committee.

Cloning was performed using somatic cell nuclear transfer as previously described [29] with donor cells from cultured ear fibroblasts obtained from a Danish Landrace × Yorkshire (65%:35%) sow. The cloned embryos were transferred surgically to surrogate sows (recipients) five to six days after cloning as described [30]. The cloned piglets were obtained by Caesarian section on gestation day 116 [30]. The sows were treated 24 h before with a prostaglandin analogue (175 μg Estrumate i.m., Pitman-Moore, UK).

As controls, normal litters (75% Danish Landrace × 25% Yorkshire) were obtained after standard artificial insemination and Caesarian section. All pigs were reared in the experimental stables of Aarhus University (Tjele, Denmark).

Two surrogate sows gave birth to 9 cloned piglets, of which 5 survived. Two normal control litters resulted in 18 female pigs, of which 6 were allocated as controls. Pigs were then nursed by surrogate sows and weaned after 28 days. They were kept on a standard diet for an additional 2 months, and were individually housed thereafter. The weight at 3 months of age was for clones 37.8 ± 4.0 kg (Mean ± SEM, n = 5) and for controls 37.9 ± 2.3 kg (n = 6). The pigs were then fed *ad libitum *with a wheat-based high energy diet containing 10% sugar and 10% soy oil. Blood from the jugular vein was taken biweekly after overnight fasting for plasma preparation. The blood samples were stored on ice and centrifuged within 1.5 h at 3000 rpm for 10 min at 4°C. Pigs were killed with a bolt pistol at an age of 8 1/2 months after overnight fasting. Weights of clones and controls at time of slaughter were 143.6 ± 8.8 kg and 179.5 ± 4.0 kg, respectively. Blood samples were obtained during desangiunation for serum preparation, and urine and bile samples were obtained directly by puncture of the respective bladders. The blood samples were placed 1 h at room temperature and subsequently centrifuged at 3000 rpm for 10 min at 4°C. Serum, plasma, bile, and urine samples were frozen and kept at -80°C until analysis.

### NMR spectroscopy

The NMR measurements were performed at 310 K on a Bruker Avance III 600 spectrometer, operating at a ^1^H frequency of 600.13 MHz, and equipped with a 5-mm ^1^H TXI probe (Bruker BioSpin, Rheinstetten, Germany).

For plasma and serum samples 500 μL aliquots were mixed with 100 μl D_2_O containing 0.05% w/w sodium trimethylsilyl-[2,2,3,3-^2^H_4_]-1-propionate (TMSP), for urine samples 300 μL aliquots were mixed with 300 μL D_2_O containing 0.005% w/w TMSP, while for bile samples 400 μL aliquots were mixed with 200 μl D_2_O containing 0.025% w/w TMSP. Urine pH was adjusted to 7.0 prior to NMR measurements. On all bio-fluids, standard one-dimensional (1D) ^1^H NMR spectra were acquired using single 90° pulse experiment with a total of 64 scans and a relaxation decay of 5 s. Water suppression was achieved by irradiating the water peak during the relaxation delay, and 16 K data points spanning a spectral width of 12.15 ppm were collected. In addition, on plasma samples 1D ^1^H NMR spectra were also acquired with a Carr-Purcell-Meiboom-Gill (CPMG) delay added in order to attenuate broad signals from high-molecular-weight components. In the CPMG experiment a relaxation decay of 3 s was applied and 32 K data points spanning a spectral width of 17.36 ppm were collected.

### Data pre-processing

All spectra were referenced to the TMSP signal at 0 ppm and the spectral region from 0.5 - 9.5 ppm was used. For multivariate data analysis spectra were aligned using the icoshift procedure [31] in MATLAB (version R2009b, The Mathworks Inc., Natick, MA, USA). Then spectra were subdivided into 0.006 ppm spectral regions and integrated, leaving out the region 5.0 - 4.6 ppm, which included residual water resonance.

Normalization is often a prerequisite for bio-fluid analysis, especially for urine, because absolute urine metabolite concentrations are highly variable. However, the normalization procedure also affects score- and loading plots in multivariate data analysis [32]. In the present study multivariate data analysis (SIMCA-P+ software, Umetrics AB, Umeå, Sweden) of spectral data was carried out both on raw data and data normalized to the total signal intensities of the NMR spectra.

### Multivariate dataanalysis

All multivariate analyses were carried out with full cross-validation (leave-one-out) and principal component analysis (PCA) was applied to the centered and paretoscaled data to explore any clustering behaviour of the samples. Furthermore, orthogonal partial least squares discriminant analysis (O-PLS-DA) was performed on spectra of the plasma samples.

Serum samples collected on the day of slaughter were included in the analysis of plasma samples. No grouping of the serum samples was observed in the PCA, and therefore plasma and serum was pooled for the multivariate analysis.

### Statistical analysis

All statistical analyses were performed using the Statistics Toolbox in MATLAB. The relative integrals from plasma analysis were analysed using a one-way analysis of covariance.

For investigation of the variability of metabolite concentrations within groups, integrals of baseline separated metabolites were determined using Topspin 2.1 (Bruker Biospin, Faellanden, Switzerland). The variances of the integrals were determined within each experimental group (clone/control). For urine, normalized integrals were used, whereas for serum and bile absolute integrals were used. In order to test the equality of variance, an F-test was carried out using the *vartest2 *function in Matlab, and p-values below 0.05 were considered significant.

For each metabolite the ratio between cloned and control variances was calculated. Values smaller than one thus reflect a lower variance in the cloned group and a value higher than one reflects a higher variance in the cloned group. For each metabolite the probability of the ratio to be either above or below one was expected to be 0.5 and binomially distributed. Thus, the probability of observing a number of ratios below one was computed using the *binocdf *function and a p-value below 0.05 was considered significant.

## Authors' contributions

JSG, HC, HCB and MSH designed the experiments. YL and HC cloned the embryos based on cells prepared by SP. MS transferred the embryos and performed the Caesarian sections. JSG, MSH, and KLC performed the animal experiment and the sampling, MRC did the NMR data analysis and assignments, statistical and multivariate data analysis and wrote the manuscript. MRC, HCB, JSG, and HC edited the manuscript, and the manuscript was read and approved by all authors.

## Supplementary Material

Additional file 1 **Compound IDs**. Manuscript compound IDs, compound names and Pubchem compound IDs. Click here for file

Additional file 2 **Raw data**. All raw data as phased, baseline corrected, normalized, and binned NMR spectra. Click here for file

Additional file 3 **Integrals**. Metabolite integrals for all individuals and F-statistics. Click here for file
