# Supplementary material for: Metabolomic phenotyping of a cloned pig model
Source: BMC Physiol. 2011 Aug 22;11:14. doi: 10.1186/1472-6793-11-14 (PMC3174869; doi:10.1186/1472-6793-11-14)
Supplement: Additional file 1 — Compound IDs. Manuscript compound IDs, compound names and Pubchem compound IDs. [file 1472-6793-11-14-S1.DOC]

Table A1. Identified compounds in plasma and urine and Pubchem compound ID, where relevant^a^

| Manuscript peak number | Pubchem compound ID | Compound Name |
| --- | --- | --- |
| 17 | 6106 | Leucine |
| 18 | 6287 | Valine |
| 19 | 6306 | Isoleucine |
| 20 | 612 | Lactate |
| 21 | 5950 | Alanine |
| 22 | 196 | Adipate |
| 23 | 176 | Acetate |
| 27 | 1060 | Pyruvate |
| 28 | 611 | Glutamate |
| 29 | 30586 | Creatine |
| 30 | 305 | Choline |
| 31 | 1145 | Trimethylamine-N-oxide |
| 32 | 5793 | glucose |
| 33 | 588 | creatinine |
| 34 | 6057 | tyrosine |
| 35 | 6140 | phenylalanine |
| 36 | 11552 | isovaleraldehyde |
| 37 | 11671 | 2-hydroxyisobutyrate |
| 39 | 311 | citrate |
| 40 | 674 | dimethylamine |
| 41 | 1146 | trimethylamine |
| 42 | 1123 | taurine |
| 43 | 750 | glycine |
| 44 | 68144 | phenylacetylglycine |
| 45 | 464 | hippurate |
| 47 | 1204 | allantoin |
| 48 | 1176 | urea |
| 50 | 764 | guanine |

^a^ only signals arising from single compounds, that are available in Pubchem are shown.
