# Supplementary material for: Metabolomic phenotyping of a cloned pig model
Source: BMC Physiol. 2011 Aug 22;11:14. doi: 10.1186/1472-6793-11-14 (PMC3174869; doi:10.1186/1472-6793-11-14)
Supplement: Additional file 3 — Integrals. Metabolite integrals for all individuals and F-statistics. [file 1472-6793-11-14-S3.DOC]

Table A2. Bile. Absolute peak areas for all individuals, standard error (standard deviation/sq. root (n-1)) for the two groups and F-statistics. Only baseline separated peaks are included in the table.

| Manuscript peak number^a^ (observed δ, ppm) | Cloned pig no | | | | | Control pig no | | | | | | Standard error | |  |  |
| --- | --- | --- | --- | --- | --- | --- | --- | --- | --- | --- | --- | --- | --- | --- | --- |
|  | 1 | 2 | 3 | 4 | 5 | 1 | 2 | 3 | 4 | 5 | 6 | Cloned pigs | Control pigs | F-value | p value |
| 1 (0.62) | 91.9 | 96.1 | 94.2 | 93.7 | 99.0 | 87.0 | 87.4 | 102.5 | 99.3 | 99.5 | 90.4 | 1.37 | 3.06 | 0.16 | 0.10 |
| 4 (2.75) | 6.4 | 9.2 | 6.8 | 9.0 | nd. | 3.3 | 2.9 | 4.3 | 5.6 | 4.7 | 5.0 | 0.84 | 0.46 | 2.00 | 0.47 |
| 5 (3.07) | 13.1 | 5.9 | 11.4 | 9.7 | 11.8 | 7.1 | 8.9 | 16.8 | 7.0 | 10.7 | 6.8 | 1.38 | 1.73 | 0.51 | 0.53 |
| 6 (3.25) | 22.9 | 31.2 | 23.1 | 30.4 | nd. | 12.3 | 12.3 | 15.6 | 19.1 | 16.7 | 18.2 | 6.15 | 1.30 | 2.40 | 0.37 |
| 7 (3.45) | 8.6 | 8.5 | 9.8 | 8.9 | 8.4 | 9.0 | 9.4 | 10.9 | 13.0 | 11.1 | 13.5 | 0.30 | 0.82 | 0.10 | 0.048 |
| 12 (4.30) | 6.1 | 8.5 | 6.3 | 7.6 | nd. | 2.7 | 2.9 | 4.5 | 5.0 | 4.4 | 4.3 | 0.65 | 0.42 | 1.41 | 0.69 |
| 12 (4.42) | 1.3 | 1.8 | 1.3 | 1.6 | nd. | 0.5 | 0.5 | 0.9 | 1.0 | 0.8 | 0.9 | 0.15 | 0.09 | 1.53 | 0.63 |
| 15 (7.80) | 14.7 | 16.7 | 15.2 | 15.6 | 16.1 | 14.1 | 13.8 | 15.2 | 18.0 | 16.6 | 16.3 | 0.40 | 0.72 | 0.24 | 0.19 |
| 15 (7.98) | 3.9 | 2.7 | 3.2 | 3.0 | 2.9 | 1.8 | 2.2 | 4.9 | 2.5 | 3.5 | 1.8 | 0.23 | 0.55 | 0.14 | 0.076 |

Table A3. Plasma. Absolute peak areas shown as means ± standard deviation over the entire sampling period for all individuals, standard error (standard deviation/sq. root (n-1)) for the two groups and F-statistics. Only baseline separated peaks are included in the table.

| Manuscript  peak number^a^ (observed δ, ppm) | Cloned pig no | | | | | | | | | | | | | | | Control pig no | | | | | | | | | | | | | | | | | |
| --- | --- | --- | --- | --- | --- | --- | --- | --- | --- | --- | --- | --- | --- | --- | --- | --- | --- | --- | --- | --- | --- | --- | --- | --- | --- | --- | --- | --- | --- | --- | --- | --- | --- |
|  | 1 | | | 2 | | | 3 | | | 4 | | | 5 | | | 1 | | | 2 | | | 3 | | | 4 | | | 5 | | | 6 | | |
| 18 (1.03) | 0.0313 | ± | 0.0204 | 0.0671 | ± | 0.0847 | 0.0279 | ± | 0.0120 | 0.0410 | ± | 0.0148 | 0.0379 | ± | 0.1207 | 0.0308 | ± | 0.0175 | 0.0374 | ± | 0.0170 | 0.0260 | ± | 0.0180 | 0.0254 | ± | 0.0113 | 0.0464 | ± | 0.0250 | 0.0349 | ± | 0.0234 |
| 20 (4.10) | 0.2667 | ± | 0.1303 | 0.2291 | ± | 0.1442 | 0.1897 | ± | 0.0725 | 0.1486 | ± | 0.0402 | 0.2363 | ± | 0.1269 | 0.1459 | ± | 0.0798 | 0.2090 | ± | 0.1646 | 0.1421 | ± | 0.0809 | 0.2069 | ± | 0.1858 | 0.2355 | ± | 0.1242 | 0.2219 | ± | 0.1231 |
| 21 (1.47) | 0.0360 | ± | 0.0228 | 0.0498 | ± | 0.0222 | 0.0321 | ± | 0.0124 | 0.0429 | ± | 0.0146 | 0.0357 | ± | 0.1244 | 0.0276 | ± | 0.0104 | 0.0378 | ± | 0.0192 | 0.0253 | ± | 0.0180 | 0.0277 | ± | 0.0140 | 0.0444 | ± | 0.0307 | 0.0325 | ± | 0.0165 |
| 23 (1.91) | 0.0211 | ± | 0.0059 | 0.0228 | ± | 0.0113 | 0.0168 | ± | 0.0053 | 0.0195 | ± | 0.0055 | 0.0220 | ± | 0.1284 | 0.0167 | ± | 0.0065 | 0.0205 | ± | 0.0057 | 0.0154 | ± | 0.0073 | 0.0164 | ± | 0.0065 | 0.0210 | ± | 0.0069 | 0.0206 | ± | 0.0066 |
| 27 (2.36) | 0.0141 | ± | 0.0065 | 0.0143 | ± | 0.0097 | 0.0102 | ± | 0.0030 | 0.0141 | ± | 0.0045 | 0.0147 | ± | 0.1319 | 0.0093 | ± | 0.0039 | 0.0126 | ± | 0.0042 | 0.0103 | ± | 0.0062 | 0.0108 | ± | 0.0036 | 0.0159 | ± | 0.0096 | 0.0124 | ± | 0.0047 |
| 33 (4.04) | 0.0130 | ± | 0.0057 | 0.0138 | ± | 0.0062 | 0.0102 | ± | 0.0038 | 0.0142 | ± | 0.0057 | 0.0125 | ± | 0.1218 | 0.0103 | ± | 0.0043 | 0.0127 | ± | 0.0085 | 0.0104 | ± | 0.0054 | 0.0091 | ± | 0.0061 | 0.0147 | ± | 0.0083 | 0.0125 | ± | 0.0068 |
| 34 (7.18) | 0.0147 | ± | 0.0054 | 0.0132 | ± | 0.0062 | 0.0108 | ± | 0.0044 | 0.0130 | ± | 0.0056 | 0.0140 | ± | 0.0066 | 0.0125 | ± | 0.0062 | 0.0148 | ± | 0.0070 | 0.0111 | ± | 0.0065 | 0.0113 | ± | 0.0061 | 0.0130 | ± | 0.0055 | 0.0137 | ± | 0.0037 |

Table A3, continued

| Manuscript  peak number^a^ (observed δ, ppm) | Standard error | |  |  |
| --- | --- | --- | --- | --- |
|  | Cloned pigs | control pigs | F-value | P-value |
| 18 (1.03) | 0.0077 | 0.0035 | 4.6 | 1.90E-08 |
| 20 (4.10) | 0.0229 | 0.0178 | 0.78 | 0.36 |
| 21 (1.47) | 0.0035 | 0.0033 | 1.02 | 0.93 |
| 23 (1.91) | 0.0012 | 0.0011 | 1.21 | 0.47 |
| 27 (2.36) | 0.0009 | 0.0010 | 1.35 | 0.25 |
| 33 (4.04) | 0.0008 | 0.0009 | 0.63 | 0.093 |
| 34 (7.18) | 0.0007 | 0.0006 | 0.94 | 0.83 |

Table A4. Urine. Relative peak areas for all individuals, standard error (standard deviation/sq. root (n-1)) for the two groups and F-statistics. Only baseline separated peaks are included in the table.

| Manuscript peak number^a^ (observed δ, ppm) | Cloned pig no | | | | | Control pig no | | | | | | Standard error | |  |  |
| --- | --- | --- | --- | --- | --- | --- | --- | --- | --- | --- | --- | --- | --- | --- | --- |
|  | 1 | 2 | 3 | 4 | 5 | 1 | 2 | 3 | 4 | 5 | 6 | Cloned pigs | Control pigs | F-value | p value |
| 21 (1.48) | 0.9 | 4.8 | 4.6 | 7.4 | 4.2 | 2.9 | 0.8 | 1.6 | 3.0 | 3.6 | 3.0 | 1.2 | 0.5 | 5.06 | 0.10 |
| 33 (3.04) | 3.7 | 126.4 | 232.7 | 478.6 | 496.8 | 172.2 | 48.7 | 44.1 | 58.1 | 220.9 | 203.6 | 108.3 | 37.1 | 6.81 | 0.06 |
| 33 (4.05) | 3.7 | 92.4 | 161.4 | 332.6 | 348.6 | 119.4 | 34.6 | 31.1 | 41.8 | 156.8 | 142.8 | 75.2 | 26.0 | 6.68 | 0.06 |
| 36 (0.94) | 1.6 | 8.6 | 7.5 | 16.3 | 17.6 | 6.3 | 2.8 | 2.8 | 4.4 | 9.7 | 8.3 | 3.3 | 1.3 | 5.35 | 0.09 |
| 37 (1.36) | 1.8 | 4.5 | 5.8 | 10.7 | 9.9 | 4.5 | 1.3 | 1.6 | 2.9 | 5.1 | 5.2 | 1.9 | 0.8 | 4.54 | 0.13 |
| 44 (7.37) | 0.2 | 6.7 | 10.4 | 12.2 | 9.4 | 5.7 | 1.8 | 3.9 | 3.1 | 8.0 | 12.6 | 2.3 | 1.8 | 1.40 | 0.71 |
| 45 (3.97) | 2.9 | 50.7 | 32.3 | 60.8 | 87.2 | 21.6 | 10.9 | 8.0 | 22.0 | 28.6 | 28.7 | 15.8 | 3.9 | 13.0 | 0.02 |
| 45 (7.55) | 2.8 | 32.2 | 14.4 | 22.3 | 46.8 | 7.6 | 6.0 | 3.7 | 13.3 | 9.4 | 9.7 | 8.4 | 1.5 | 25.7 | 0.003 |
| 45 (7.64) | 3.4 | 16.3 | 8.1 | 14.2 | 28.2 | 5.1 | 3.1 | 2.0 | 6.7 | 6.7 | 6.5 | 4.7 | 0.9 | 21.5 | 0.005 |
| 45 (7.84) | 2.3 | 30.5 | 14.1 | 19.5 | 40.3 | 7.2 | 6.0 | 3.6 | 12.4 | 8.9 | 9.5 | 7.3 | 1.4 | 23.3 | 0.004 |
| 46 (7.06) | 3.0 | 27.4 | 31.4 | 63.5 | 68.2 | 5.9 | 2.2 | 6.9 | 5.6 | 33.9 | 33.6 | 13.5 | 6.6 | 3.33 | 0.22 |
| 46 (7.23) | 3.5 | 17.3 | 26.3 | 56.8 | 60.3 | 1.9 | 0.7 | 5.9 | 4.0 | 32.2 | 27.5 | 12.4 | 6.3 | 3.15 | 0.24 |
| 47 (5.39) | 2.1 | 4.3 | 15.1 | 31.2 | 31.4 | 13.8 | 1.9 | 2.8 | 2.0 | 18.2 | 16.8 | 7.1 | 3.5 | 3.24 | 0.23 |
| 48(5.78) | 1.6 | 252.6 | 319.1 | 647.9 | 400.8 | 290.9 | 124.6 | 78.2 | 167.3 | 326.1 | 395.1 | 117.2 | 56.0 | 3.51 | 0.20 |
| 49(5.08) | 5.2 | 6.3 | 7.9 | 20.3 | 22.1 | 0.0 | 0.1 | 1.9 | 0.7 | 10.3 | 8.1 | 4.1 | 2.0 | 3.23 | 0.23 |
